# New jumping spiders from the alpine meadows of the Valley of Flowers, western Himalayas, India (Araneae, Salticidae)

**DOI:** 10.3897/zookeys.783.25225

**Published:** 2018-09-05

**Authors:** John T.D. Caleb, S.K. Sajan, Vikas Kumar

**Affiliations:** 1 Centre for DNA Taxonomy, Zoological Survey of India, Prani Vigyan Bhawan, M-Block, New Alipore, Kolkata-700053, West Bengal, India Zoological Survey of India Kolkata India

**Keywords:** *
Nandicius
*, *
Pellenes
*, new species, Uttarakhand, taxonomy

## Abstract

Two new jumping spider species: *Nandiciusvallisflorum***sp. n.**, (♀) and *Pelleneshimalaya***sp. n.** (♂, ♀), are diagnosed, described and illustrated in detail. The specimens were collected from the Valley of Flowers, western Himalayas, Uttarakhand State of India.

## Introduction

The Indian salticid diversity is represented by 245 species in 79 genera (WSC 2018) of which, 65 species under 40 genera are known from across the Indian Himalayan region (Caleb and Kumar 2018a). While examining specimens from recent biodiversity surveys on the alpine meadows of the ‘Valley of Flowers’ (VOF), a high-altitude Himalayan valley in Uttarakhand state of India, two new species of jumping spiders were recognized. The VOF is a recognized UNESCO world heritage site known for its unique natural elegance, and is home to several rare, endangered, and high-altitude flora and fauna (Kala 2005).

The genus *Nandicius* Prószyński, 2016 contains eight valid species from Asia, of which two species, *N.frigidus* (O. Pickard-Cambridge, 1885) and *N.mussooriensis* (Prószyński, 1992) are known from India (WSC 2018). The genus *Pellenes* Simon, 1876 consists of 83 described species known from Asia, Africa, Europe, North America and Australia (WSC 2018). Two species are known from India, *P.allegrii* Caporiacco, 1935 and *P.maderianus* Kulczyński, 1905 (Caporiacco 1935; Prószyński 1992). The record of *P.maderianus* in India is presumably doubtful and misidentified as stated by both Logunov et al. (1999) and Prószyński (2016). Recently, a new species, *P.iva* Caleb, 2018 was described from South India (Caleb and Kumar 2018b). The present paper provides detailed descriptions and illustrations of two new species: *Nandiciusvallisflorum* sp. n., and *Pelleneshimalaya* sp. n. This paper is part of an on-going study recording the biodiversity across the Indian Himalayan region.

## Materials and methods

Specimens were hand collected and preserved in 70% ethanol. Morphological examination and photography were performed under a Leica EZ4 HD stereomicroscope. Detailed micro-photographs were obtained using a Leica M205A stereomicroscope attached with Leica DFC500 HD camera enabled with a Leica Application Suite (LAS) version 3.8. Epigyne was dissected and macerated in 10% KOH to clear soft tissue. Temporary preparations were observed and photographed using a Leica DM1000 compound microscope attached with a Leica EC3 camera. Line drawings were prepared with the GNU Image Manipulation Program (GIMP) (Montesanto 2015). All measurements are in millimetres; leg measurements are given in the following order: total (femur, patella, tibia, metatarsus, tarsus). Spine positions are as follows: prolateral, dorsal, retrolateral and ventral. The type specimens are deposited in the National Zoological Collections, Zoological Survey of India (**ZSI**), Kolkata.

Abbreviations used in the text are as follows:

**AER** anterior eye row;

**ALE** anterior lateral eye;

**AME** anterior median eye;

**CBP** central blind pocket;

**CTA** compound terminal apophysis;

**FL** eye field length;

**PER** posterior eye row;

**PLE** posterior lateral eye;

**PME** posterior median eye;

**RTA** retrolateral tibial apophysis.

## Taxonomy

### Family Salticidae Blackwall, 1869

#### Genus *Nandicius* Prószyński, 2016

##### Nandicius vallisflorum sp. n.

Taxon classificationAnimaliaAraneaeSalticidae

http://zoobank.org/19EA84C0-6D7A-477C-8C93-FF5737BB3D91

Figs 1–4 , 5–8

###### Holotype.

Female (ZSI-CDT-AA1649) from Valley of Flowers (30.72362°N, 79.58764°E), 3567 m a.s.l., Chamoli District, Uttarakhand, India, 16 June 2017, S.K. Sajan & party.

###### Etymology.

The specific name is derived from the location ‘valley of flowers’ (‘vallis florum’ in Latin).

###### Diagnosis.

Female of *N.vallisflorum* sp. n. can be distinguished from other congeners except *N.mussooriensis* (Prószyński, 1992) in having an epigyne with crescent shaped sclerotized margins within which the copulatory openings are present (cf. Figs 5, 7 with fig. 58 in Prószyński, 1992); it can be distinguished from *N.mussooriensis* by the following characters: posterior rim of epigyne sclerotized with a wide protrusion bearing wide epigynal pockets on either sides positioned between the spermathecae and the curved sclerotized margins (protrusion absent and pockets widely placed directly below spermathecae in *N.mussooriensis* (cf. fig. 59 in Prószyński, 1992 with Figs 6, 8); copulatory openings present medially in the oval depressions (positioned posteriorly in *N.mussooriensis*) (Figs 5, 7); proximal region of spermathecae pear-shaped (stomach-shaped in *N.mussooriensis*) (Figs 6, 8).

###### Description.

**Figures 1–4.**
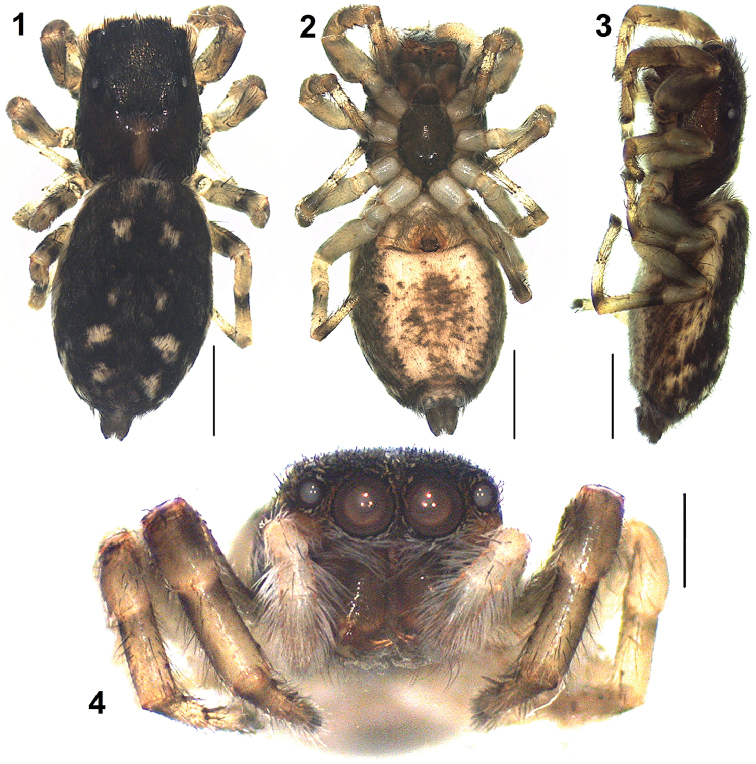
*Nandiciusvallisflorum* sp. n., holotype female. **1** general appearance, dorsal view **2** same, ventral view **3** same, lateral view **4** front view. Scale bars: 1 mm (**1–3**), 0.5 mm (**4**).

**Figures 5–8.**
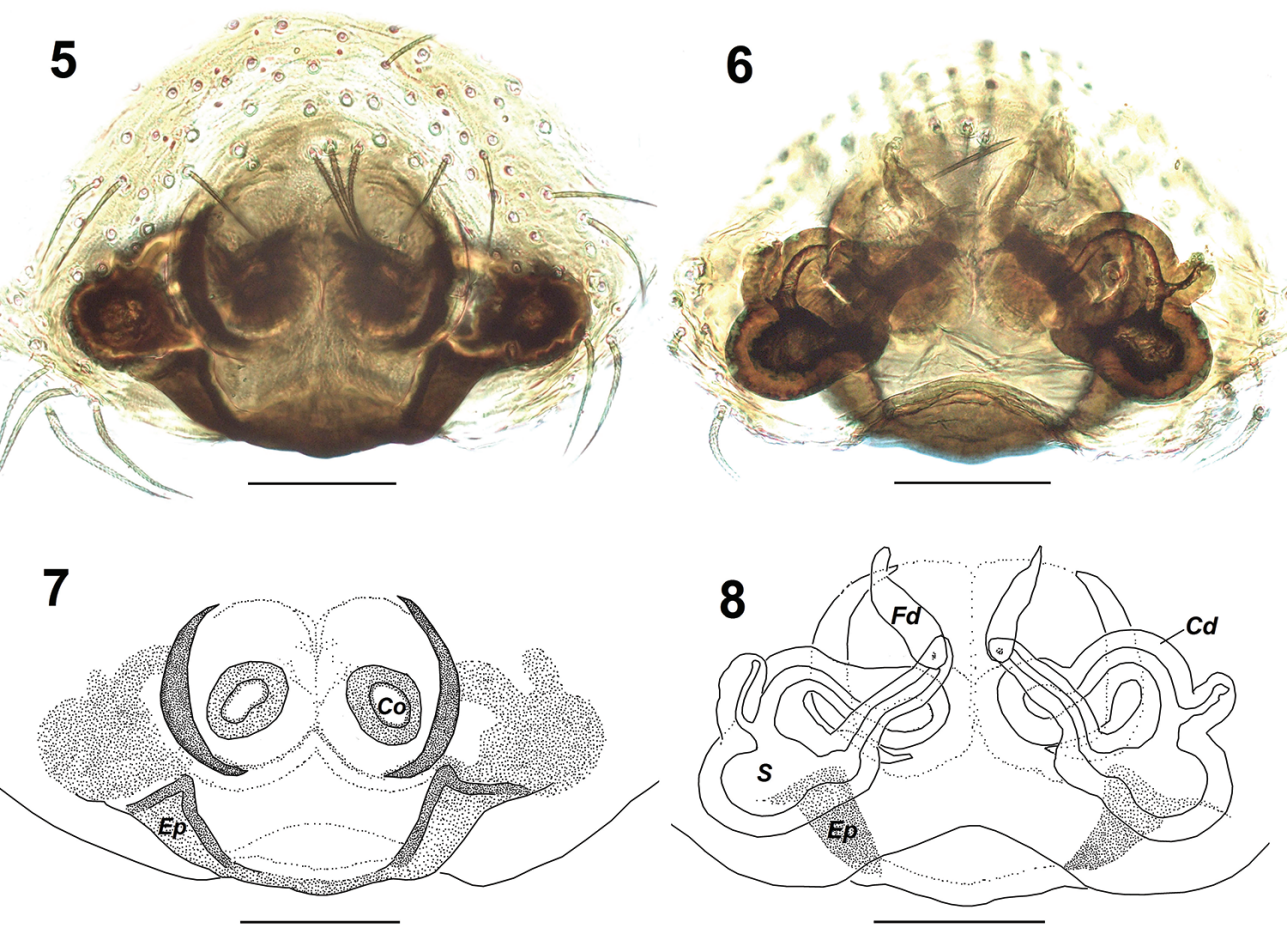
*Nandiciusvallisflorum* sp. n., holotype female. **5** epigyne, ventral view **6** vulva, dorsal view **7** epigyne, ventral view **8** vulva, dorsal view. Abbreviations: Co – copulatory opening; Cd – copulatory duct; Ep – epigynal pocket; Fd – fertilization duct; S – spermatheca. Scale bars: 0.1 mm.

*Female* (holotype). Total length: 4.59; carapace: 1.87 long, 1.45 wide; abdomen: 2.74 long, 1.84 wide. Carapace dark brown, with a light brown median patch present at the posterior region; the outer edge of carapace covered with pale white setae (Figure 1). Eye field covered with black hairs and pale white scales. Anterior eyes surrounded by reddish-brown orbital setae except for the lower margin lined with white setae; clypeus covered with long white hairs (Figure 4). Eye measurements: AME 0.34, ALE 0.15, PME 0.05, PLE 0.16, AER 1.12, PER 1.15, EFL 0.75. Clypeus height 0.05. Sternum oval, dark brown. Chelicerae reddish-brown, unidentate; labium and maxillae brownish (Figure 2). Legs yellowish with dark brown annulations on the segments (Figs 1–3, 4); palps yellowish clothed with dense white hairs. Leg measurements: I 2.93 (0.92, 0.59, 0.60, 0.43, 0.39); II 2.67 (0.85, 0.58, 0.48, 0.40, 0.36); III 2.92 (0.91, 0.49, 0.52, 0.55, 0.45); IV 3.69 (1.12, 0.55, 0.80, 0.74, 0.48). Leg formula: 4132. Leg spination: femora I 0400, II 0300, III 0300, IV 0300; patellae I–IV 0000; tibiae I 2006, II 1001, III 2011, IV 1013; metatarsi I 3005, II 1004, III 3021, IV 2021; tarsi I–IV 0000. Abdomen blackish with a pale white chevron shaped marking mid-dorsally accompanied by pairs of yellowish-white spots present longitudinally (Figure 1); lateral margins yellowish (Figure 3); venter with brown lateral region and broad central yellowish region covered with irregular brown spots (Figure 2). Spinnerets brownish (Figs 1, 2). Epigyne with copulatory openings located in oval depressions in the medial region separated by a thin median septum; outer, lateral rim sclerotized and crescent-shaped (Figs 5, 7); copulatory ducts diverge laterally, reaching the spermathecae; spermathecae with pear-shaped proximal portion and elongated distal portion (Figs 6, 8).

*Male.* Unknown.

###### Distribution.

India (Uttarakhand).

#### Genus *Pellenes* Simon, 1876

##### Pellenes (Pelmultus) himalaya sp. n.

Taxon classificationAnimaliaAraneaeSalticidae

http://zoobank.org/A5D6D940-A002-4C69-9C13-5EDB19B103BB

Figs 9–13 , 14–19 , 20–24 , 25–27 , 28–30

###### Holotype.

Male (ZSI-CDT-AA1636) from Valley of Flowers (30.71142°N, 79.59615°E), 3256 m a.s.l., Chamoli District, Uttarakhand, India, 10 June 2017, leg. Pritam Kumar Dey.

###### Paratypes.

3 females (ZSI-CDT-AA1644, 1637, 1638); 3 males (ZSI-CDT-AA1645 to 1647) and 2 subadults (ZSI-CDT-AA 1639, 1648) from same location, 10.06.2017, leg. S.K. Sajan, Pritam Kumar Dey & Soumyasree Sen.

###### Etymology.

The specific name is a noun in apposition, referring to the great Himalayan Mountain range from where the species was collected.

###### Diagnosis.

**Figures 9–13.**
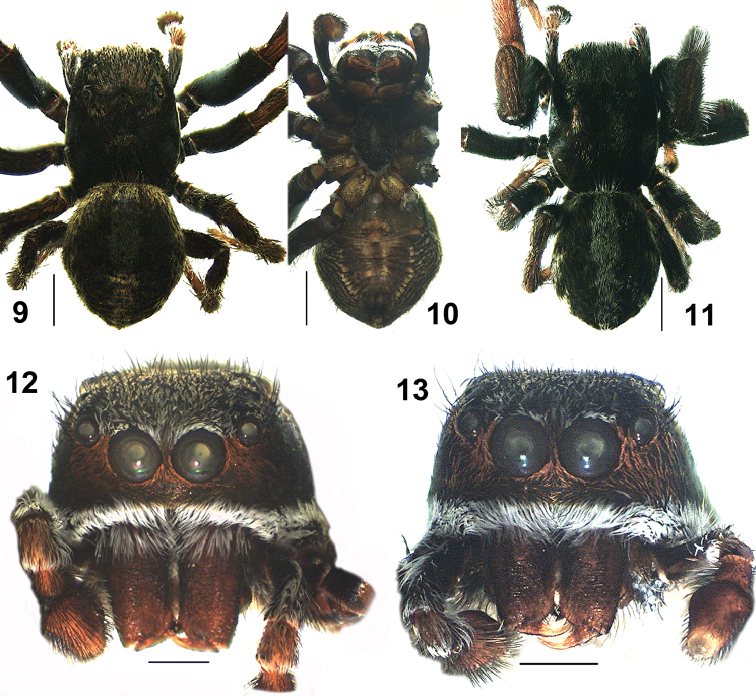
*Pelleneshimalaya* sp. n. **9–11** general appearance. **9** dorsal view (holotype) **10** ventral view **11** dorsal view of paratype (AA1646). **12–13** carapace, front views. **12** holotype **13** paratype (AA1645). Scale bars: 1 mm (**9–11**); 0.5 mm (**12–13**).

The males of *P.himalaya* sp. n. can be readily distinguished from other congeners except *P.allegrii* Caporiccao, 1935, *P.pamiricus* Logunov, Marusik & Rakov, 1999 and *P.bitaeniata* (Keyserling, 1882) in having a similar embolic division and CTA. From *P.allegrii* it can be distinguished by the RTA directed at 12 o’ clock position (curving dorsally and directed at 1 o’ clock position in *P.allegrii*; cf. Figs 22, 23 with figs 69, 81 in Logunov et al. 1999); from *P.pamiricus* by the wider cymbial lobe, covering the entire RTA in dorsal view (protruding ventrally, covering the basal part of RTA in *P.pamiricus*; cf. Figure 24 with fig. 185 in Logunov et al. 1999); from *P.bitaeniata* by the lack of terminal protrusion of CTA and the larger cymbial lobe (CTA terminally elongated and cymbial lobe indistinct in ventrolateral view; cf. Figs 20, 21, 26 with figs 8, 9 in Żabka 2006). In addition, *P.himalaya* sp. n. can also be separated easily from other congeners by the relatively longer and slender tibia and metatarsus of leg I (Figure 27). Females can be easily distinguished from other species by the distinctly long and conical CBP and closely placed spermathecae (Figs 28, 30).

###### Description.

*Male* (holotype AA1636). Total length: 5.74; carapace: 2.88 long, 2.28 wide; abdomen: 3.07 long, 2.43 wide. Carapace black, covered with black hairs and leaf-like dull yellowish scales (Figs 9, 11–12). Anterior eyes surrounded by rusty-brown orbital setae except for the top margin with white setae. Clypeal region covered with reddish-brown hairs below the anterior eyes reaching to the ‘cheek region’. Dense white hairs forming a thick white band clothe the front edge of the carapace and runs along the entire rim. White hairs present on the basal quarter of the chelicerae (Figs 12–13). Eye measurements: AME 0.45, ALE 0.23, PME 0.05, PLE 0.21, AER 1.55, PER 1.63, EFL 1.15. Clypeus height 0.21. Sternum oval, blackish (Figure 10). Chelicerae reddish-brown, unidentate; labium and maxillae dark brown with paler outer margins. Legs with blackish femora and brown patellae, tibiae and metatarsi, except yellow-brown tibia I (Figs 9, 11, 27); all tarsi light brown; femur I with ventral fringe of white setae (Figure 27). Leg measurements: I 9.08 (2.55, 1.52, 2.40, 1.72, 0.89); II 5.10 (1.64, 0.99, 1.02, 0.80, 0.65); III 5.80 (1.92, 1.07, 1.16, 1.09, 0.56); IV 5.67 (1.79, 0.90, 1.14, 1.16, 0.68). Leg formula: 1342. Spination. Legs: femora I 0400, II 0600, III 0600, IV 0300; patellae III–IV 1010; tibiae I 1006, II 1006, III 3034, IV 3024; metatarsi I 0004, II 2004, III 3034, IV 4043; tarsi I–IV 0000. Abdomen ovoid, blackish; covered with black hairs and yellowish scales. A broad median stripe of white hairs present, which continue as chevron shaped markings posteriorly; anterior margin and lateral margins outlined with pale white hairs (Figs 9, 11); ventral region brownish, with a pair of light brown longitudinal patch; spinnerets brownish (Figure 10). Pedipalps light brown except blackish femur; femur and patella covered with white hairs and scales (Figs 12–13, 24); embolus tapering, with a thin tip, accompanied by a CTA; RTA thick and strong with a blunt tip; cymbial lobe distinct protruding retrolaterally (Figs 20–26).

**Figures 14–19.**
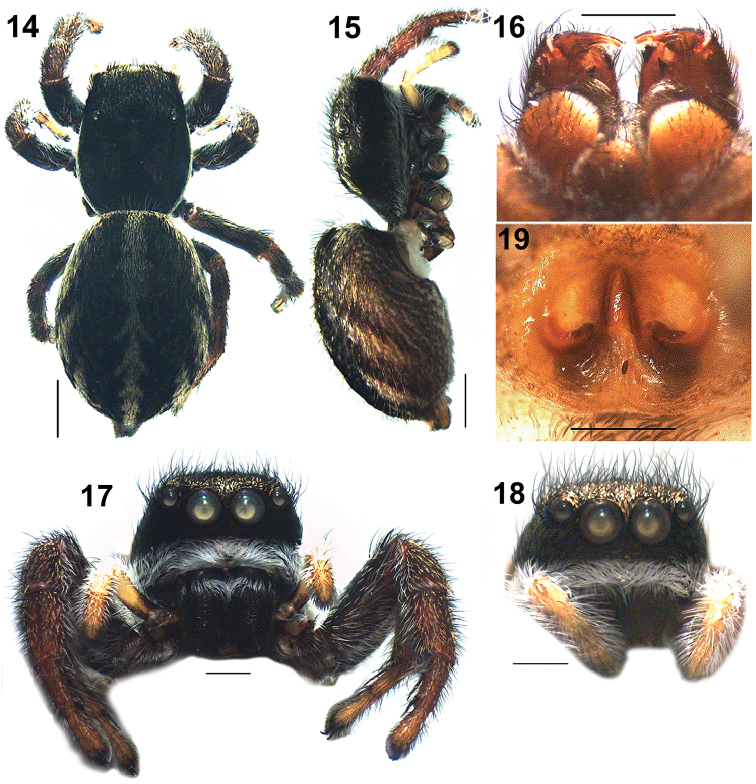
*Pelleneshimalaya* sp. n., **14–17** paratype female (AA1644). **14** general appearance, dorsal view **15** same, lateral view **16** chelicerae, ventral view **17** front view **18** front view of paratype (AA1638) **19** epigyne, ventral view of paratype (AA1638). Scale bars: 1 mm (**14–15**); 0.5 mm (**17–18**); 0.2 mm (**19**).

*Female* (paratype AA1644). Total length: 6.59; carapace: 2.65 long, 2.11 wide; abdomen: 3.97 long, 2.86 wide. Eye measurements: AME 0.44, ALE 0.24, PME 0.08, PLE 0.22, AER 1.49, PER 1.55, EFL 1.03. Clypeus height 0.35. Leg measurements: I 4.69 (1.43, 1.00, 0.96, 0.73, 0.57); II 4.01 (1.29, 0.88, 0.72, 0.59, 0.53); III 5.11 (1.65, 0.97, 0.98, 0.89, 0.62); IV 5.32 (1.67, 0.85, 1.06, 1.01, 0.73). Leg formula: 4312. Spination. Legs: femora I 0500, II 0500, III 0500, IV 0300; patellae I 0000, II 1000, III 1010, IV 1000; tibiae I 0006, II 0004, III 3023, IV 3024; metatarsi I 0004, II 0004, III 3034, IV 4043; tarsi I–IV 0000. Coloration pattern as in male (Figs 14–18). Chelicerae with two fused teeth on promargin and one tooth on the retromargin (Figure 19). Pedipalps yellowish clothed with dense white hairs (Figs 17–18). Epigyne with a long, conical CBP and lateral crescent shaped openings (Figs 19, 28); internal structures shown in Figs 29, 30.

**Figures 20–24.**
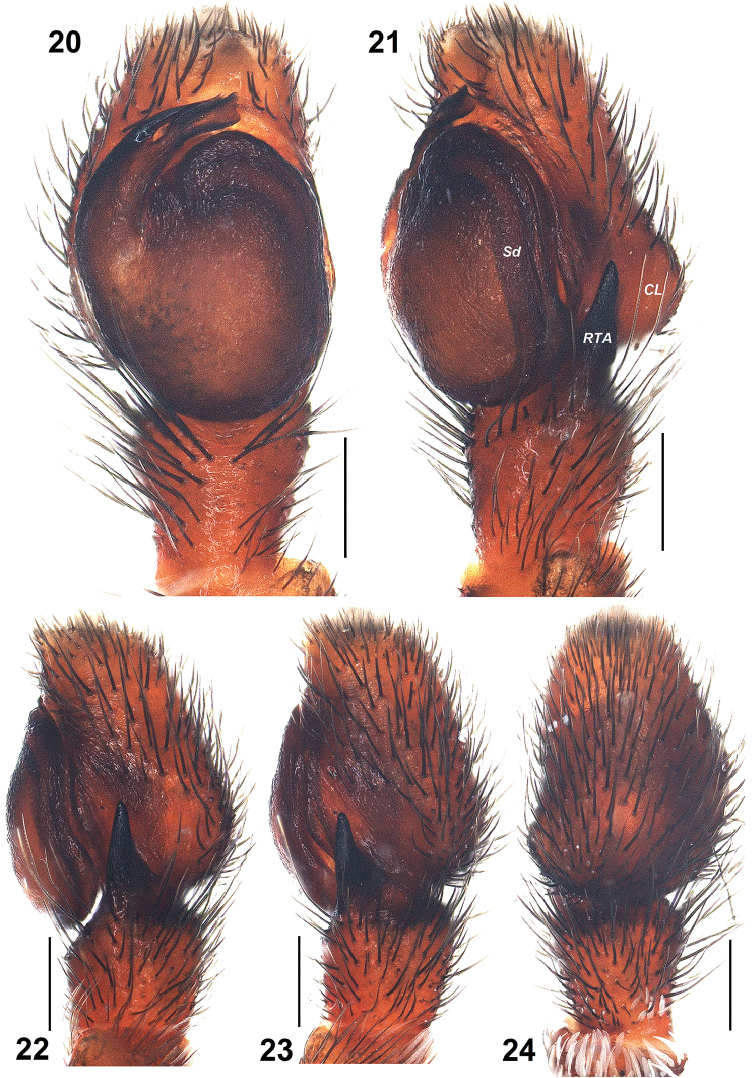
*Pelleneshimalaya* sp. n., holotype male. **20** male left palp, ventral view **21** same, ventro-lateral view **22**, same, retrolateral view **23** same, dorso-lateral view **24** same, dorsal view. Scale bars: 0.2 mm (**20–24**). Abbreviations: CL – cymbial lobe; RTA – retrolateral tibial apophysis; Sd – sperm duct.

**Figures 28–30.**
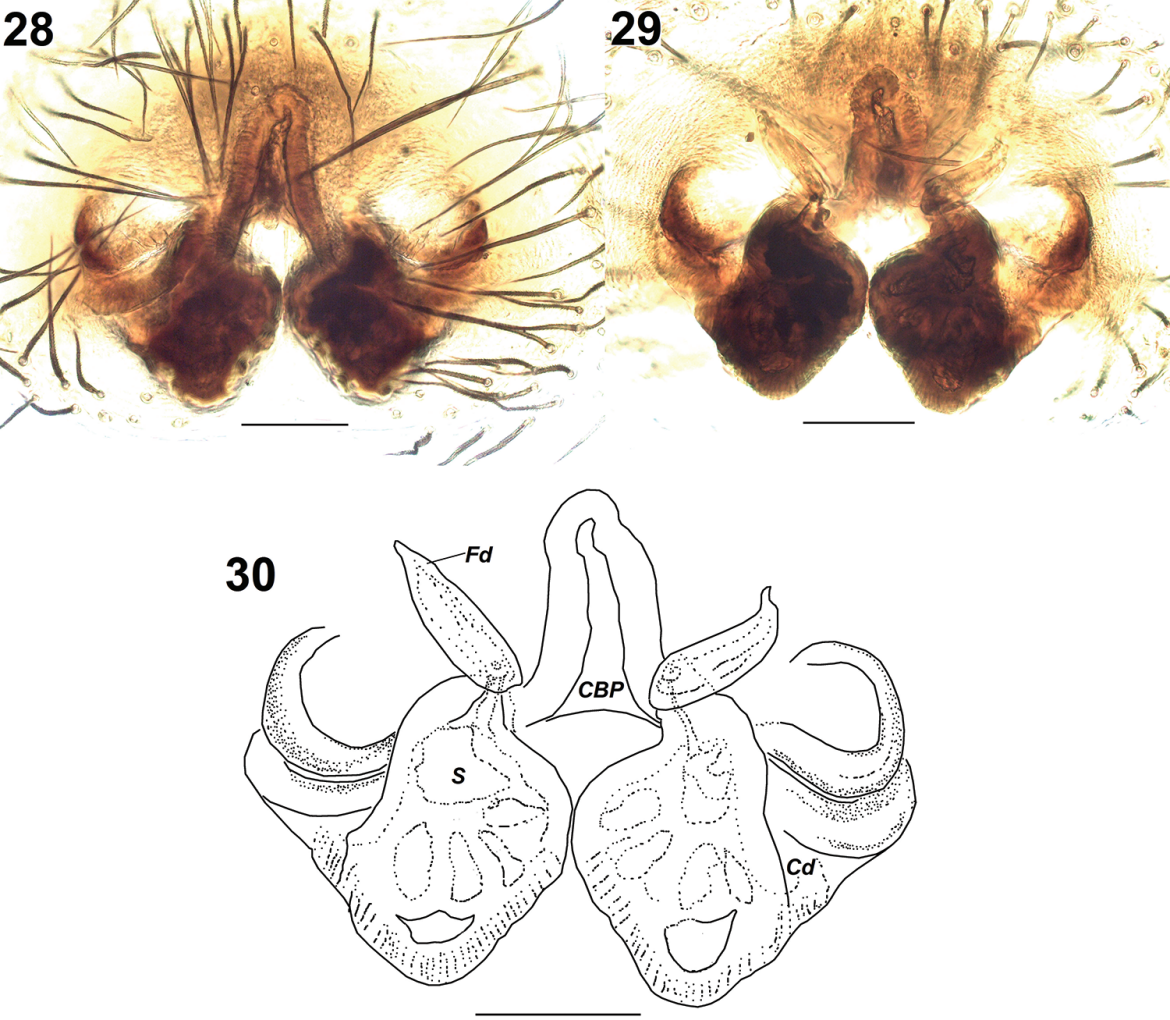
*Pelleneshimalaya* sp. n., paratype (AA1644). **28** epigyne, ventral view **29** vulva, dorsal view **30** same. Abbreviations: CBP – central blind pocket; Cd – copulatory duct; Fd – fertilization duct; S – spermatheca. Scale bars: 0.1 mm (**28–30**).

**Figures 25–27.**
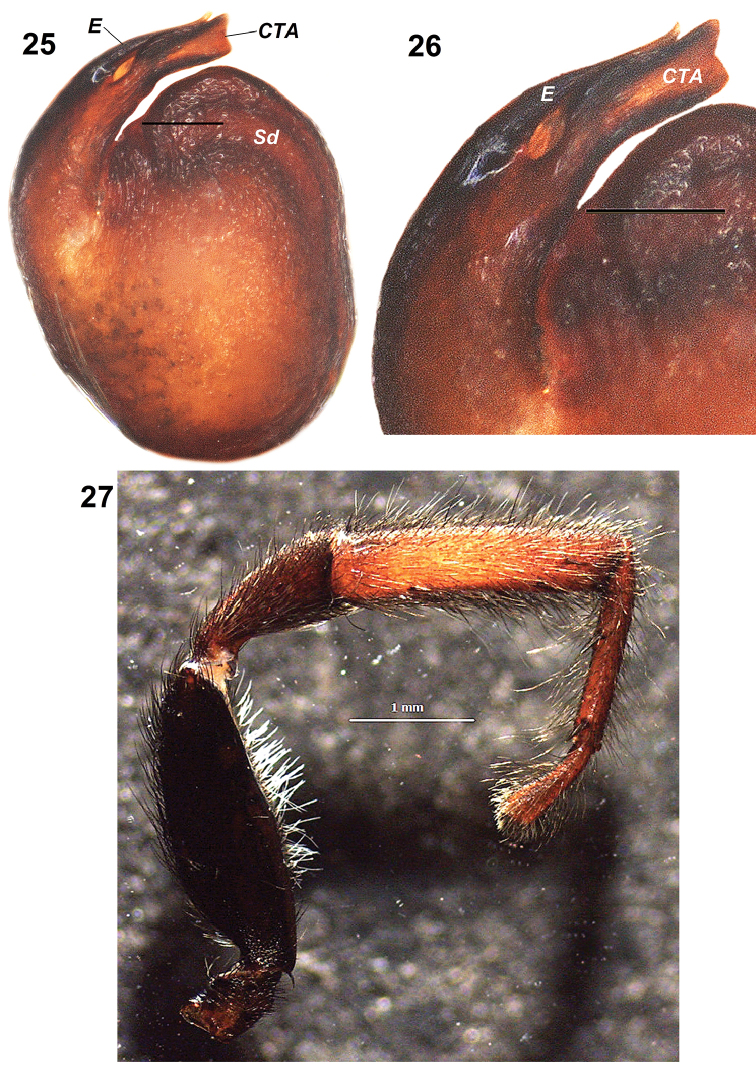
*Pelleneshimalaya* sp. n., holotype male. **25** male left palp, ventral view **26** same, ventro-lateral view **27** left leg I, prolateral view. Scale bars: 0.1 mm (**25–27**). Abbreviations: CTA – compound terminal apophysis; E – embolus; RTA – retrolateral tibial apophysis; Sd – sperm duct.

###### Distribution.

India (Uttarakhand).

###### Variation.

Body length: Male: 5.04–5.89 (n = 4). Female: 5.47–6.71 (n = 3). The thickness of the band of white hairs on the base of the chelicerae is variable within both males (Figs 12, 13) and females (Figs 17, 18).

###### Natural History.

**Figure 31.**
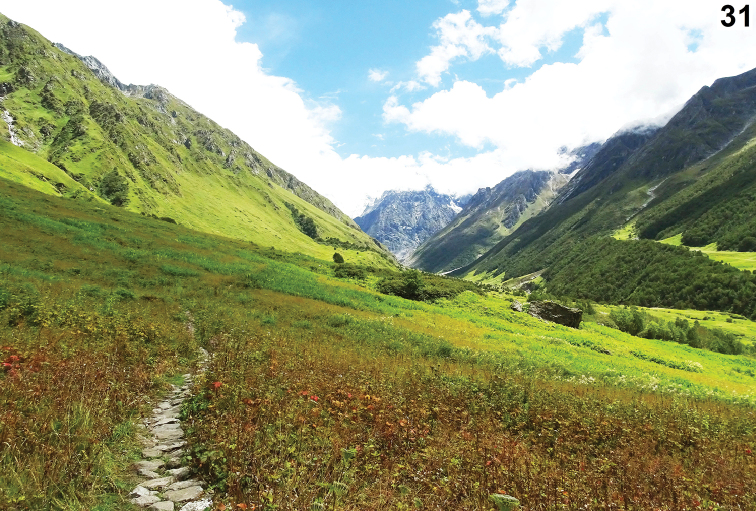
Alpine meadow habitat of *Nandiciusvallisflorum* sp. n. and *Pelleneshimalaya* sp. n. Image was kindly provided by Dibyajyoti Ghosh.

Both the new species were collected from the alpine meadows of the VOF (Figure 31) at altitudes ranging between 3200 to 3600 meters above mean sea level. The heterogeneous landscape ranges from the low-lying flat and gentle slopes to steep and high snow-bound areas, providing a great diversity of microhabitats resulting in a rich diversity of both flora and fauna (Kala 2005).

## Supplementary Material

XML Treatment for Nandicius vallisflorum

XML Treatment for Pellenes (Pelmultus) himalaya
